# The value of experts by experience in social domain supervision in the Netherlands: results from a ‘mystery guests’ project

**DOI:** 10.1186/s12913-024-10692-y

**Published:** 2024-02-09

**Authors:** Sophia M. Kleefstra, Brenda J.M. Frederiks, Adriënne Tingen, Petra G.J. Reulings

**Affiliations:** 1 Dutch Health and Youth Care Inspectorate, Utrecht, the Netherlands; 2 Amsterdam UMC, department Ethics, Law and Medical Humanities, Amsterdam, the Netherlands; 3 Department of patient care, University Medical Centre Groningen, Groningen, the Netherlands

**Keywords:** Experts by experience, Patient and public involvement, participation, healthcare supervision, Healthcare regulation, Quality improvement, Persons with intellectual disabilities

## Abstract

**Background:**

User involvement and participation in the supervision of the quality of care is an important topic for many healthcare inspectorates. It offers regulators an additional view on quality, increases the legitimacy and accountability of the inspectorate, empowers users and enhancing the public’s trust in the inspectorate. To assess the accessibility of the local governmental social domain services the Joint Inspectorate Social Domain in the Netherlands worked together with people with intellectual disabilities performing as ‘mystery guests’ in an innovative project. This paper describes the findings of the evaluation of this project.

**Methods:**

People with intellectual disabilities living at home on their own may need some help with daily activities such as administrative tasks, raising children, household tasks, managing debts or finding work. In the Netherlands they have to arrange this help at their municipality. The goal of this project was to find out how easily people with intellectual disabilities could get help from their municipality. The participants were equal partners with the JISD inspectors from the beginning: in constructing an inspection framework, in acting as mystery guest with a fictive support request, reported back the results by storytelling.

**Results:**

The evaluation of the project showed that the JISD succeeded in their key aspect of the project: the goal to involve people with intellectual disabilities in a leading role from the beginning until the end. Their perspectives and preferences were the starting point of supervision. Pain points in accessibility became clear straight away and gave important insights for both inspectors as municipality professionals. Municipalities started to improve their services and evaluated the improvements with the clients. Furthermore, the impact on the participants themselves was also huge: they felt being taken seriously, valued and empowered.

**Conclusion:**

Involving people with intellectual disabilities as participants in all phases of supervision processes contributes to more relevant and useful outcomes, creates mutual understanding of perspectives, as affirmed by both municipalities and inspectors, and creates empowerment of the participants. Furthermore, it fits perfectly within the United Nation Convention on the rights of persons with disabilities and the current development of ‘value driven regulation’.

**Supplementary Information:**

The online version contains supplementary material available at 10.1186/s12913-024-10692-y.

## Background

Since several years user involvement and participation in the supervision of the quality of care is an important part of the supervision agenda of many healthcare inspectorates [1]. Quality of care from the users perspective offers regulators an additional view, alongside the traditional inspection’s perspective of quality and safety, often based on laws, guidelines and protocols [1–4]. It also increases the legitimacy and the accountability of a regulator, empowers the users by giving them a voice in supervision [1, 5, 6] and therefore enhancing the public’s trust in the inspectorate [7].

Involving users has been a theme on the multi sequential annual policy agendas of the Dutch Health and Youth Care Inspectorate (DHYI) from 2016 until 2023. There have been a lot of initiatives to hear the users voice about quality of care, some singular pilots, some in a structural way [1]. The users perspective is for instance structurally included in risk based supervision by means of reviews of a patient rating website [3, 8], in citizen’s panels focusing on specific themes, in complaint handling by the inspectorate [5, 9] and in inspections by means of the observation tool Short Observation Framework for Inspections (SOFI), consultations and interviews [10].

Also two pilots were performed with experts by experience as mystery guests [11] and as lay inspectors accompanying inspectors in supervising elderly care organizations [7]. An expert by experience is a person who is in between the life-world of users and that of the system of professional inspectors and policymakers [12] and should therefore be able to be a bridge between the two worlds [7]. The added value of these pilots involving experts by experience as mystery guests or lay inspectors in regulation inspections is clear, for instance providing information complementary to that gathered by inspectors [11]. However, their practical wisdom, providing subjective and experiential ways of knowing instead of objective measurable aspects of knowing [7], has also been criticized during the evaluation of the pilots.

The evaluation of these pilots with inspectors and other stakeholders showed points of attention to reflect on when involving experts by experience in supervision. Resistance to external, political, pressure to incorporate users perspective was a complicating factor in the first pilot with the mystery guests. Moreover, inspectors had concerns about practical problems and barriers. For instance existing procedures and formats assessing quality and safety aspects which didn’t fit with experiential information of other, softer aspects of care obtained from users. Also contrasting notions or what constitutes valid reviews and reports hindered the use of the experiences of the experts by experience as mystery guests in daily practice [11]. Furthermore, in the lay inspector’s pilot the methodology of the pilot structured the experts by experience in such a way that their knowledge was unlikely to be opened up. For instance, much effort was made to select the ‘proper’ clients: the selection criteria for the experts by experience resulted in verbally strong, well-acquainted in the healthcare system and actively involved people with limited personal experiences. They took the oath as (unpaid) public officials, which gave them a formal status. Their training focused on providing unbiased, objective observations, emphasizing their professional roles as public officials. Moreover, the experts by experience were not involved in creating the themes for the interviews, nor the structure of the written report. Their input was used as illustrations instead of relevant input. Finally, the professional way of knowing was repeatedly valued more than the practical wisdom of the experts [7].

Although involving experts by experience in supervision is seen as a highest form of a participatory regulation method [13], the DHYI invested a lot of effort in these pilots and learned a lot, it still remained a challenging quest. The main challenge seemed to incorporate the experiences and knowledge of the experts by experience in existing supervision processes. Their input was often given less credibility, or disregarded in the end [7, 11]. This issue of marginalizing the knowledge of people is often referred to as ‘epistemic injustice’: doing wrong to someone in their capacity as a knower [14, 15]. In inspection practice it means for instance that users experiential knowledge is granted of less value than inspectors professional knowledge, and a greater credibility is given to inspectors than to peoples assessment of the quality of care [1, 16, 17]. The integration of different types of knowledge is experienced as complicated [18]. Objections, such as the subjectivity of the experiential knowledge, occur when perspectives on quality of healthcare differ, or even oppose [2, 3]. Assessing, or improving care from the users perspective, is often hindered by this phenomenon [1]. However, it is not only applicable to inspectors, or health professionals in healthcare organizations [14, 19–23]. In fact, epistemic injustice is often an unwittingly and common phenomenon in all our lives [15]. Hence, it is a real barrier to actually value a different kind of knowledge, such as the users experiential perspective in daily supervision, healthcare improvement, complaints or incident investigation [1, 23–25].

In an attempt to counter the challenges from the earlier pilots with mystery guests and lay inspectors, the Joint Inspectorate Social Domain (JISD) in the Netherlands performed a new mystery guest project in 2017. The goal of the project was to assess the accessibility of the local governmental social domain services for people with intellectual disabilities living at home on their own, for instance whether the information was given in an easy language or in easy read. At the same time the JISD wanted to shed light on the question in which situations and conditions these experts by experience could add value to the inspectorate’s daily supervision. This project was evaluated by an independent academic research organization, focusing on the value of this mystery guests method and using the users perspective in assessing the accessibility of the social domain services [26].

In this paper we report the findings of the evaluation of this Accessibility project. In the methodology section we describe the project itself and the method of evaluation. The Guidance for Reporting Involvement of Patients and the Public (GRIPP2) is used to report the results [27]. The results section consists of the results of the evaluation from the perspectives of the people with intellectual disabilities, inspectors and the municipalities. In the discussion part we interpret the challenges from the two earlier pilots and reflect on the added value of this project for the use of experts by experience in daily supervision practice. Furthermore we make recommendations when using the mystery guests method for supervision.

## Methods

### The accessibility project

In the Netherlands about 1.1 million people have intellectual disabilities [28, 29]. It is often not visible. Most people with intellectual disabilities live at home on their own. However, they may need some help with daily activities such as administrative tasks, raising children, household tasks, managing debts or finding work. They have to arrange this help at their municipality by themselves. Often, they are not recognized as vulnerable people and therefore they may not get the help they really need. The United Nations Convention on the rights of persons with disabilities however, ensures persons with disabilities for instance of full and effective participation in society on an equal basis with others and full access to society [30]. Together with people with intellectual disabilities the JISD assessed in 2017 the accessibility of the social domain services of five municipalities in the Netherlands. The goal of this Accessibility project was to find out how easily people with intellectual disabilities could get help from their municipality.

The JISD is a partnership of four governmental inspectorates: the Health and Youth Care Inspectorate, the Inspectorate of Education, the Inspectorate of Justice and Security and the Inspectorate of Social Affairs and Employment. JISD’s inspections are mainly theme based, focusing on public social problems concerning young people or vulnerable adults [4].

Based on the experiences and recommendation of the English regulator of health and adult social care CQC [29] with experts by experiences, the JISD collaborated throughout the project with a client association, the LFB [31]. The LFB is a national client association for and by people with intellectual disabilities, to support and empower them to be able to participate in Dutch society. The LFB participated in selecting people and in the coaching of people by someone well-known and trusted during the whole project. Together with inspectors the LFB set up a training for the participants and their coaches. In four sessions of one day, four groups of people with intellectual disabilities, their coaches and inspectors got to know each other, received information about the project and practiced a lot. For instance, they performed role plays with a ‘public servant’ behind a table asking questions to the participants. They learned about the questions the inspectors would ask after the visits. Furthermore, they learned about role of the coach. The coaches were available for the participants from the beginning of the project, during and afterwards. They knew the participants very well and acted as trusted sparring partners and joined the participants during their mystery visits. They were introduced as ‘the neighbor’ or ‘the brother/sister’ of the participant. However, he or she was preferably not supposed to join conversations at the mystery visit. And afterwards save their opinion after the mystery guest had spoken out their experiences. Furthermore, the coaches provided support and aftercare when necessary. The training learned the inspectors to connect with the participants and coaches in language and attitude. The participants and coaches learned what was expected of them in the role of a mystery guest or a coach.

Participants of the five municipalities were city councilor, service manager or public servant. The city councilor authorized the mystery visits at the municipality and two of them were handed over the report afterwards. The service managers joined the feedback meetings and indicated the improvements to be made. The public servants underwent the mystery visits and reacted to their experiences.

The people with intellectual disabilities were involved as equal partners in the project from the beginning. First of all, they helped inspectors constructing an inspection framework. Before the start of the project JISD organized together with the LFB a meeting with 16 people with intellectual disabilities to inventorize aspects that consider most to them in contact with the municipalities. The aspects were prioritized in importance by the participants themselves. Examples of these aspects were: to use an easy language, to listen carefully to the question asked and to be patient in contact. Subsequently, in co-operation between inspectors and participants, the existing framework was adapted to the perspective of the people with intellectual disabilities. Important issues were added, such as the check of the public servant whether people really understood the received information. However, as a consequence, aspects not mentioned or not important for the participants, for instance the formal requirement for municipalities to offer client support, were deleted from the framework. Inspectors had to let go of their own vision on accessibility and their existing framework.

Second, the JISD elaborated fictive questions based on the legal tasks of the municipalities, for instance offering support with educational issues, with domestic services, with managing debts or to find a job. Several teams of two people with intellectual disabilities, together with their coach and 2 inspectors, assessed the websites of two different municipalities at the same time, trying to answer questions such as ‘Can I get support in raising my children?’. Assessing the same question for two municipality websites showed the differences between the websites. The results were written down in a paragraph in the municipality reports.

Third, the participants acted as mystery guests and visited their municipality with a fictive support request and assessed the service they received. The participants chose request topics that suited their circumstances most or they felt familiar with. Once the participants had chosen a support topic, this request was ‘rebuilt’ based on their experiences or knowledge, and was well discussed with their coach until they felt comfortable with it. Then they started the mystery visit, accompanied by their coach.

Fourth, all the experiences of the mystery guests with a municipality were collected by the inspectors. Subsequently, the JISD organized two moments of feedback. The first meeting was between the people with intellectual disabilities and the public servants involved. The participants narrated their experiences: what went well and what could be better? The public servants reacted: did they recognize the experiences? This resulted in a dialogue between the participants and public servants. What were the good points to keep and what could be improved? The second meeting was between inspectors and the service managers of the municipality. The inspectors reported the experiences of the mystery guests, and the reactions of the public servants. The managers indicated important improvements, prioritized these in easy to do and necessary improvements. In both meetings the conclusions were drawn by the municipality professionals– not by the JISD. This supervision method is called ‘feedback without recommendations’ [32]. Because the improvements were formulated by the professionals themselves instead of the inspectorate, they generate an intrinsic motivation to improve.

Fifth, after the meetings with the public servants and managers of the municipalities, the JISD produced their final reports. The experiences of the mystery guests were written down in a storytelling and comprehensible way in easy language. The recommendations to improve derived from the professionals and people with intellectual disabilities involved completed some of the reports [33]. The final reports were offered to the councilor or the city council of the municipality by the participants, in presence of the JISD.

And finally a website was built, in accordance with the participants, where non-participating municipalities could find tips to improve the accessibility of their social domain services [34].

In 2018 this project won the Dutch Innovation in Regulation Prize [35]. A motivation given by the jury: ‘by approaching the problems from the view point of people with intellectual disabilities the analyses get clear and the solutions tangible. It shows courage to involve this group as mystery guests and as a supervising authority appeal to the intrinsic motivation of municipalities to improve instead of waving with a raised finger’.

### The evaluation

The evaluation of the Accessibility project was conducted in April 2018-April 2019 by the Amsterdam Public Health academic research institute of the VU medical centre [26]. The research team consisted of two senior researchers (BF and AT) and a research assistant. A supervisory committee, consisting of seven experts with academic and professional background and a member of the participating client organization LFB, supported the researchers at three moments in the evaluation. This independent evaluation started after the project was finished. It consisted of a qualitative approach, combining interviews with a literature quick scan and a document analysis.

The literature quick scan based on predefined terms was conducted to provide an overview of the results of the use of experts by experience pilots for supervision purposes, both national and international. The document analysis consisted of analyzing all documents of the project, such as the project plan, the assessment framework and the meeting reports, and the final reports for the municipalities. Based on the quick scan and the document analysis the researchers pointed out five new elements in the Accessibility project. A topic list for the interviews was constructed based on these results.

In the period of June-October 2018 the researchers conducted 28 semi-structured interviews based on a qualitative method of analysis [36]. Nine people with intellectual disabilities and their coaches, one city councilor, six service managers and public servants of the municipalities, and three inspectors were interviewed. All interviews were transcribed verbatim and anonymized. The researchers and the research assistant independently coded the transcripts in open, inductive coding. After coding the first seven interviews, the research team merged the codes, identified themes and discussed to reach consensus (axial coding). After that the coding continued and the themes were sharpened when necessary. The last step was connecting themes together in overarching themes (selective coding). These overarching themes were discussed with the participating people with intellectual disabilities. The results from the literature quick scan, the document analysis and the interviews were discussed with the supervisory committee in three meetings.

The final evaluation report described the results from the literature scan and the document analysis, concluding that the project was innovative in five new elements. Subsequently, the central part of the evaluation report consisted of describing these five innovative elements based on the interviews, supplemented by a conclusion and recommendations for the future.

## Results

We describe the result section by focusing on the five innovative elements. These elements cover the key aspect of the project: involving the people with intellectual disabilities from the beginning until the end of the project and take their perspective as the starting point for supervision.

### Added value of people with intellectual disabilities as expert by experience

The people with intellectual disabilities involved in the project valued the huge amount of personal attention by the inspectors before the start. The support of the accompanying coach was valued as indispensable. The coaches however revealed that the time invested was much more than expected at the beginning. Furthermore, the participants indicated that they had more insight in the work of the inspectors, and a better understanding of the processes and procedures at their municipality. This would facilitate the next contact with their municipality. Being involved in this project had empowered them. They indicated a higher self-confidence, felt they were taken seriously and valued. Also being able to represent other people with intellectual disabilities in an important project from the beginning was experienced as valuable, especially because this project could really make a difference for people with intellectual disabilities. The LFB finally established a bigger foothold in some municipalities.

‘*I received a certificate, (…) well, that was great. (…) I was kind of proud of myself*’ (participant, interview 15).

‘*It made me a little bit stronger, and also, that I dare to do more*’ (participant, interview 2)

‘(…) *It is told by the people themselves and not by their family, or by their guide or brothers, sisters. People look at it differently if it is told by people themselves, because it has more impact if people with intellectual disabilities tell it themselves*’ (participant, interview 13).

Municipalities were given more insight in and awareness of the impact of their service on the people with intellectual disabilities and what they need in contact with their municipality. In particular practical matters such as how they are addressed, the intelligibility of public servants and whether the municipality uses accessible language and pictograms. Being assessed by people with intellectual disabilities opened the eyes of the public servants to things they never even thought of themselves. For instance, a website specifically developed for people with intellectual disabilities that could not be found by them, or a town hall without pictograms so people experienced this entrance very unclear and confusing. Furthermore, interviewed public servants revealed that hearing what can be improved from the people with intellectual disabilities themselves had far more impact than when the same things were told by the inspectorate.

‘*And using the experiences of these people [with intellectual disabilities] points the finger on the sore spot immediately: where do you fail or what can be improved?*’ (public servant, interview 11).

The councilor added that the information obtained from the participants can be seen as honest and sincere. Their testimonies were seen as ‘what you see is what you get’, tackling the risk of only saying what you wanted to hear. Also the risk of being ‘institutionalized’ will probably be less a problem with most people with intellectual disabilities. Focusing on expressing their personal experiences instead of training on being a ‘professional mystery guest’ prevented the institutionalization.

Also the inspectors were given more insight in experiences of people with intellectual disabilities and the impact of municipal services on them. The project was rather small-scale, with five participating municipalities, and therefore the inspectors were very involved in the process. In most cases they sat next to the participants contacting the municipality by phone or e-mail. They experienced almost literally what impact the service of the municipality had on the participants. This approach provided the inspectors with knowledge that would not have been revealed in a conventional inspection. For instance, the impact of not being addressed properly by a public servant or being confronted with a telephone menu can result in not sleeping well at night, relating the lack of service to themselves, or panicking and disconnecting the phone, not daring to contact the municipality again.

These elements, such as the way people were being addressed and approached in a personal way, which are essential for people with intellectual disabilities, were usually not part of an inspection framework. In general, inspection frameworks consist for instance of standards of safe and responsible care and/or have a focus on compliance with laws and rules. However, due to this project the inspectors became more aware of the importance of user experiences.

‘*We witnessed most of what the participants experienced because we were there and sat alongside while they were calling; and you could see at their faces oh this is not working out well and things like that cannot be written down in a questionnaire or so’* (inspector).

### Developing an inspection framework together with people with intellectual disabilities

Adapting a supervision framework was facilitated by the work field of the social domain. The JISD focusses on to help find options to deal with social problems that are themes of the inspections, in this project the accessibility of municipal services for people with intellectual disabilities. There were no standards for accessibility to start from. In case of enforcement supervision, with minimum standards for safe and responsible care, and a focus on compliance with rules and laws, an inspection framework probably won’t be easily adapted in a way that the perspective of the users serves as the starting point. After all, the aspects mentioned by users often differ from those of the inspectorate. This additional view of users can therefore be difficult to fit in existing frameworks and is as a consequence often disregarded as irrelevant for enforcement supervision.

*‘We really choose from the beginning of the project for the users perspective. This was the starting and central point of the project, and the inspection framework: not what we thought was important, but what they think is important. We therefore left out items from the framework that were not mentioned by the participants, for instance about the employee who supports clients in contact with municipalities. He is very important in accessibility, but if they don’t mention him, we leave him out. Otherwise it is not their inspection framework’* (inspector).

### People with intellectual disabilities as mystery guests

When visiting the municipality the people with intellectual disabilities involved had to have a fictive support request. During the training it turned out that a support request which could be experienced by the participants themselves is the most successful. In fact, it proved to be necessary for a credible performance as a mystery guest. Another important condition was that the participant is capable of performing as a mystery guest. It requires a certain ability of abstraction, some self-confidence and mental resilience not everybody had. Furthermore, they had to be able to express their experiences into words. Therefore the LFB, who knew the participating candidates very well, had the important task to select capable candidates. Training subsequently supported and improved their capability to perform as a mystery guest. The participants appreciated the preparation by means of role plays and training. This boosted their self-confidence even more and they felt prepared to be a mystery guest. In addition, the LFB also had an important role in preparing, supporting and coaching the participants during the project. The coaches of the LFB were well-known and trusted by the participants and were experienced in dealing with people with intellectual disabilities. Furthermore, aftercare turned out to be very important for the participants. A negative experience with a municipality could have a big impact on someone’s wellbeing. At the beginning of the project there were some doubts whether the mystery guests would be influenced by their coaches. He or she perhaps would behave otherwise in contact with a public servant in the presence of their coach. This argument was tackled by the fact that in real life people with intellectual disabilities are also often accompanied by a representative. Moreover, this turned out to be a way of checking whether the public servant talked with or about the mystery guest, another important principle of the UN Convention.

Not only the position and the wellbeing of the mystery guests is important, also the impact of the confrontation with a mystery guest on the wellbeing of the public servants needs attention. This aspect of the project was underexposed in the project. The participating municipalities chose to keep the mystery guest visits to be secret for their employees. The impact on the labor law relationship of being confronted with a mystery guest must not be underestimated. For instance, the comments of the mystery guests can be included in performance reviews. Some public servants felt that they have been fooled and not being taken seriously.

*‘So it would have been nice if someone had asked me: how did you feel? What were your experiences during this day (…)?’* (public servant, interview 12).

Furthermore, for the public servant the support request needs to match with the mystery guest standing in front of them. A strange, unrealistic question had as a consequence that afterwards the experiences were taken less serious by the public servants.

*‘So I felt that some colleagues were not taken this very seriously, also because it was a very strange question that was posed. And that is definitely a pity, because you focus on that question rather than focusing on what and how to communicate with people with an intellectual disability’* (public servant, interview 17).

The embedding of the project within the municipality needs attention on the forehand, as well as the possible consequences for the labor law relationship, for instance consequences of the confidentiality for public servants, or the right to be heard. These process agreements should consists of for instance aspects of confidentiality (yes or no, on the forehand or not, and on what management level within the municipality), the consequences of the (legal) position of professionals, the planning and the way of reporting of the results to the public servants. This project demonstrated a variety of process agreements and lacks of uniformity between the municipalities. Because of the lack of strict process agreements within the project, some steps were unclear. This lead to the fact that some employees were informed, where others were not. This resulted in mixed feelings about dealing with mystery guests.

Also the amount of time between the mystery guest visits and the feedback to the public servants must be limited. If the public servant still remembers the visit and his or her reaction to the support request, this enlarges the impact. The same applies for the final report. The less time between the visits and the report, the higher the impact.

### The feedback meetings: direct dialogue between people with intellectual disabilities and municipality professionals

Participants were proud to report their experiences themselves to the municipality employees. Furthermore, the impact of the stories expressed directly to the public servants was high. Some were literally eye openers. This applies to both the good and the bad experiences. It concerns for instance the influence of manner of speaking on the participants. A participant waited literally for hours in a chair next to the phone to be called back. After all, the public servant had said: ‘I’ll call you back’. Necessary however, was an confirmed appointment in day and time to call back, preventing people waiting for hours next to the phone. Furthermore the obstacles of modern communication methods, such as a complex telephone menu or a website in a too difficult language. Finally, the need for personal contact and help from the servants. Without the individual stories of the participants these eye openers would not have been visible. This resulted for instance in several improvements municipalities made: a special desk for people with intellectual disabilities, letters which were checked and rewritten by people of the LFB or organizing a training for public servants to recognize people with intellectual disabilities.

However, some public servants experienced a feeling of unsafety being confronted with the mystery guests in a plenary feedback session with colleagues. This fact must not be underestimated and should therefore be part of the process agreements made on forehand. Therefore, in future projects with mystery guests servants need to be informed in person before and not during a plenary session. Preferably with the focus on ‘what can you learn?’, so that the right to a fair hearing is guaranteed when criticizing their performance.

### Storytelling in comprehensible language as final report

The report consisted of a short description of the project and the framework used, followed by the personal experiences and feelings of two mystery guests on their visit to the municipality and the websites. Subsequently, the response of the municipality: did they recognize the stories and what improvements will follow up? The report ends with the conclusions and recommendations. Writing a short report in a comprehensible way was also a challenge for the inspectorate. However, also the JISD should practice what they preach.

## Discussion

The JISD succeeded in their key aspect of the project: the goal to involve people with intellectual disabilities in a leading role from the beginning until the end. Their perspective and preferences were the starting points for supervision. The project definitely sheds light on the preferences and experiences of the participants in their contact with the municipalities, an important insight for municipality professionals and inspectors. By involving them during the whole project, from start to end, and literally walk behind them through the building, sit next to them when they pick up the phone or check a website, pain points, such as incomprehensible letters, difficult telephone menus or websites, unclear signs, become clear straight away. This even points out issues that inspectors did not even think of or thought of in a different way. It shows clearly the influence of attitude and manner of speaking on the participants, and the obstacles they encounter, such as the selection menu by phone, unclear or no pictograms in a big town hall, difficult language and complex websites. As a result of the project the municipalities started to improve their services and evaluated their improvements with the LFB. For instance, the LFB looked at the written letters whether they were readable and understandable for people with intellectual disabilities. And last but not least, the unexpected side effect was the huge impact on the participants themselves: they gained more self-esteem, they felt being taken seriously and valued and felt empowered by being involved in this project. It is therefore necessary that inspectorates in future projects value their involvement by financial rewards and for instance a certificate.

However, the results of this project were merely small and practical improvements in accessibility rather than long term changes. Nevertheless, one municipality hired someone with an intellectual disability to involve this perspective in policy making. Also other municipalities started their own mystery guests projects, based on the recommendations from this project. Furthermore, the project generated a lot of public and political attention, for instance by winning the Dutch Innovation in Regulation Prize 2018 [35]. Still, long term improvements in for instance the structural use of easy language or more personal contact require more attention than a mystery guest visit alone.

Concerning the concept of ‘epistemic injustice’ Fricker shows that the ability of experts by experience to be involved in a valuable way depends on their credibility and intelligibility [15, 37]. Looking at the objections and barriers from the two earlier pilots within DHYI to involve experts by experience we might recognize issues concerning these credibility and intelligibility injustices [7, 11]. Two forms of epistemic injustice are distinguished: testimonial and hermeneutical [15, 25].

Testimonial injustice indicates that certain groups have less credibility and others have a credibility excess, for instance inspectors. This is the case when negative stereotypes or prejudices play a role, such as ‘persons with intellectual disabilities are not credible reporters of their own experiences’. Although users may be very knowledgeable, their voice is not considered as a valid source of information. They may not be identified as credible knowers [38]. Therefore, their testimonies are seen as less reliable. First of all it is important to notice that assessing the quality of care by a supervision authority is supposed to be objective and measurable, often expressed in quantified parameters that warrant impartiality and justice, with a high level of credibility. Hence, the first barrier to cross is the prejudice that user testimonies are just the opposite: subjective, anecdotal, express an individual experience and are therefore difficult to use in supervision [3]. The anecdotal reporting style, with stories of individual users, environmental aspects such as color, artwork and decoration of the first pilot in the elderly care was discounted by the inspectors as being useful for regulatory review because it failed to meet their information needs. This prevented them from incorporating this information into the existing supervision dossiers. Inspectors found it too difficult to distill the signs that might be relevant to warrant action from the narrative information [11]. A way to tackle this supposed subjectivity of the experts by experience in the second elderly care pilot was their training prior to inspection visits: focused on the abilities to provide unbiased, objective observations of client’s everyday lives in care homes [7]. The JISD project however, purposively put the focus on the individual, subjective stories of the people with intellectual disabilities. In feedback meetings they narrated their personal experiences and feelings to the public servants. Furthermore, the final reports were based on a story telling way and in comprehensible language. Throughout all phases the JISD kept focusing on the subjective experiences of the participating people with intellectual disabilities, and therefore tackled testimonial injustice.

Hermeneutical injustice refers to the gap in collective interpretive resources, that causes a disadvantage when it comes to making sense of someone’s experiences [15, 37]. This means that the experiences of people with intellectual disabilities are understood by the interpretative framework of inspectors without intellectual disabilities. This intelligibility deficit is for instance shown in the selection process of the experts by experiences in the second elderly care pilot: the strict criteria resulted in participants with limited personal experiences as a client, and therefore limited experiential knowledge. Furthermore, their practical wisdom appeared to manifest primarily in their relationship building strategies with clients instead of participating in the supervision methodology [7]. The evaluations of the two pilots in the elderly care showed that the professional way of knowing is repeatedly valued more than the practical wisdom of the experts by experience [7, 11]. In fact, the inspectors in the first pilot saw no added value at all for the inspectorate of the information provided by the mystery guests. This was strengthened when the views opposed with those of the inspectors: the inspectors indicated that the information provided by the mystery guests was inconsistent, incomplete and/or incorrect [11]. Another project, concerning assessments and views on quality of care of inspectors and adolescents showed that in the case of opposed views none of the inspectors changed their judgement based on the views of adolescents. That is based on the assumption that adolescents in need of care are vulnerable people, and inspectors know what is best for this group [17]. The JISD project however tackled this consequences of hermeneutical injustice by making the aspects important for people with intellectual disabilities the focus points of the supervision framework. Furthermore, the experiences of the participants made the inspectors aware of the experiential knowledge that had never been revealed in a conventional inspection visit. Given this focus of the project there was no discussion afterwards about the added value of the participants experiences. In this way justice is done to the experiential knowledge of the participants and therefore the concept of ‘epistemic injustice’ is countered.

Since then, the JISD and the cooperating inspections such as the DHYI, increasingly involve the user’s perspective in their supervision activities. The inspectorates practice with other methods of involving people, such as patient journeys or action research together with clients. Meanwhile almost all inspection frameworks include more or less the user perspective, thus including themes that are important for the people who use the services. The knowledge and experiences of involving users and their perspective in supervision are exchanged in collaborations and (inter)national networks such as the Supervision and regulation Innovation Network for care (SINC) [1].

This project fits perfectly within the United Nations Convention on the rights of persons with disabilities, on that they should have the opportunity to be actively involved in decision making processes about policies and programs, including those directly concerning them [30]. It also fits perfectly in the current value driven development of supervision: creating awareness of the experienced gap between desired and current practice together with the people involved, which leads to the readiness for change and stimulates quality improvement [39]. With involving the people in determining the object of supervision, in the collection of data, in the interpretation and in the reporting this project can be seen as an example of ‘value driven regulation’. This means that the driver behind regulatory activities should always be creating societal values or contributing to aspired values, in this particular case a good accessibility of municipalities for people with intellectual disabilities [40].

### Recommendations for future projects in supervision with experts by experience and/or the mystery guest method

The JISD project demonstrated that it is possible to involve experts by experience in every stage of a supervision project. It showed that important conditions for successful involvement are defining the goal of involving experts by experience and their role in every phase beforehand. The focus points of the supervision framework consisted of the aspects most important for people with intellectual disabilities. In feedback meetings participants exchanged their experiences themselves with the public servants. Furthermore, the final report was based upon a story telling way and in comprehensible language.

It is evident that involving experts by experience in supervision is facilitated when the user group is specific and distinctive, and also the topic of supervision is specified, for instance a specific theme. Moreover, when inspectors shift their focus from enforcement supervision with a focus on compliance with laws and rules to tackling social problems, they have to involve relevant others [4]. Furthermore, it is helpful to have the freedom to innovate and to experience within the organization. This project did not have to fit within existing inspection processes and standards. The JISD is a small organization with short lines between inspectors and management, where everybody kept each other focused on the user perspective. It is particularly suitable for supervision methods without existing minimum standards of safe and responsible care, or compliance with rules and laws, such as the ‘feedback without recommendations’ methods of supervision. Also inspectors need to be open and motivated [41] to try new methods such as the user perspective as central point for supervision, instead of their own regular methods of (enforcement) supervision. However, even within enforcement supervision methods the user perspective can add elements that are not originally part of the inspection framework.

Using the ‘mystery guests’ -method is not suitable in every supervision project. The mystery guest method requires a large time investment, for the municipalities, the coaches and the inspectors, and trust, effort and patience in working together with a specific client group. In future mystery guest projects it is recommended to work with a real support request instead of a fictive one, for both the credibility of the participants and their ‘case’. This adds weight to the experiences of the participants and will be taken more seriously by the professionals. Also process agreements, made in advance with all people involved, need to be included in the project plan. For instance, informing professionals or not, the method of reporting and the planning. The most important learning point from this project was that it is necessary to keep a close eye on the impact and (legal) consequences of a mystery guest visit for the professionals. Therefore, it is necessary to involve the perspective of the professionals in all stages of the project and to anchor this in the project plan as well. Although the method of ‘mystery guests’ has not been used since because of the time consuming character, nowadays, with care and services that become more and more digital, it would be interesting to consider repeating it. Not only with people with intellectual disabilities but also with other groups in society who may have difficulties with digital contact, such as elderly people, people with a low literacy or with another cultural background.

## Conclusions

The JISD Accessibility project showed that involving people with intellectual disabilities as participants in all phases of supervision processes contributes to more relevant and useful outcomes, creates mutual understanding of perspectives, as affirmed by both municipalities and JISD inspectors, and it creates empowerment of the participants and their client association. It also tackles epistemic injustice by granting the leading role in the project to the participants. Therefore it fits perfectly within the United Nation Convention on the rights of persons with disabilities and the current development of ‘value driven regulation’.

### Electronic supplementary material

Below is the link to the electronic supplementary material.

Supplementary Material 1

Supplementary Material 2

Supplementary Material 3

Supplementary Material 4

## Data Availability

The datasets generated and/or analyzed during the evaluation study are not publicly available to maintain the anonymity and confidentiality of the participants, but may be available from the corresponding author on reasonable request. All other data are publicly available online and cited in the manuscript and in the reference list.

## Abbreviations

CQC: Care Quality Commission
DHYI: Dutch Health and Youth care Inspectorate
JISD: Joint Inspectorate Social Domain
LFB: Landelijke Federatie Belangenverenigingen Onderling Sterk
SOFI: Short Observation Framework for inspections
UN: United Nations
