# Supplementary material for: The value of experts by experience in social domain supervision in the Netherlands: results from a ‘mystery guests’ project
Source: BMC Health Serv Res. 2024 Feb 9;24:187. doi: 10.1186/s12913-024-10692-y (PMC10858591; doi:10.1186/s12913-024-10692-y)
Supplement: Supplementary file 1 — Supplementary Material 1 [file 12913_2024_10692_MOESM1_ESM.docx]

**GRIPP2 Short Form checklist**

**Aim: the aim of the study**

To assess the accessibility of the local governmental social domain services the Joint Inspectorate Social Domain (JISD) in the Netherlands worked together with people with intellectual disabilities performing as ‘mystery guests’. The goal of the research project was to find out how easily people with intellectual disabilities could get help from their municipality.

**Methods: description of method used for PPI in study**

The participating people with intellectual disabilities were equal partners in the project from the beginning. They helped inspectors constructing an inspection framework, judged websites of the municipalities and acted as mystery guests. They visited their municipality with a fictive support request and assessed the service they received. Furthermore, the participants reported the results themselves in a plenary session with public servants, management and councilors from the municipality, assisted by the inspectors. The results were written down in comprehensible language based on ‘story telling’, centered around the experience of the participants. Together with the inspectors they offered the reports to the municipalities. A website was built in accordance with the participants where non-participating municipalities could find tips to improve the accessibility of their social domain services. During the whole project the participants were assisted and coached by a client association. In 2018 this project won the Dutch Innovation in Regulation Prize.

**Study results: outcomes – results of PPI in study, both positive and negative**

The key aspect of the research project was involving the perspective of the participating people with intellectual disabilities in all phases of the supervision process: as starting point for supervision, in the inspection itself by acting as a mystery guest, and in the reporting phase by storytelling their experiences. It was the first time people with intellectual disabilities were, as expert by experience, part of a supervision project.

Positive outcomes were:

- The participants indicated that they had more insight in the work of the inspectors and a better understanding of the processes and procedures at their municipality. This would facilitate their future contact with the municipality;
- Participating had empowered them, they indicated higher self-confidence, they felt they were taken seriously and valued;
- Their client organization established a bigger foothold in some municipalities;
- Municipalities were given more insight in and awareness of the impact of their service on the people with intellectual disabilities and what they need in contact with their municipality;
- Being assessed by people with intellectual disabilities opened the eyes of the public servants to things they never even thought of themselves;
- Interviewed public servants revealed that hearing what can be improved from the people with intellectual disabilities themselves had far more impact than when the same things were told by the inspectorate;
- The councilor added that the information obtained from the participants can be seen as honest and sincere, instead of saying what people want to hear;
- The inspectors were given more insight in experiences of people with intellectual disabilities and the impact of municipal services on them;
- This approach provided the inspectors with knowledge that would not have been revealed in a conventional inspection;
- These elements, such as the way people were being addressed and approached in a personal way, which are essential for people with intellectual disabilities, were usually not part of an inspection framework. The existing framework was adapted to the perspective of the people with intellectual disabilities. Important issues were added, and aspects not mentioned or not important for the participants, were deleted from the framework. Inspectors had to let go of their own vision on accessibility and their existing framework;
- The experiences of the mystery guests were written down in a storytelling and comprehensible way, the recommendations to improve derived from the professionals and participating people with intellectual disabilities completed the report. Writing a report in a comprehensible way was also a challenge for the inspectorate.

Negative outcomes were:

- Not everyone with intellectual disabilities can perform as a mystery guest in a supervision project. A person needs to have a certain ability of abstraction, self-confidence and mental resilience. Also the support request should match with the person performing as mystery guest. A training prior to, and support and coaching during the performance, is necessary. This helps preventing negative experiences, which could have a big impact on someone’s wellbeing
- The impact of being confronted with a mystery guest must not be underestimated. Some public servants felt that they have been fooled and not being taken seriously. Also some public servants experienced a feeling of unsafety being confronted with the mystery guests in a plenary feedback session with colleagues.

**Discussion and Conclusion: outcomes – comment to the extent to which PPI influenced the study overall, describe positive and negative effects**

The JISD succeeded in their key aspect of the project: the goal to involve people with intellectual disabilities in a leading role in supervision from the beginning until the end. Their perspective and preferences were the starting points. The project definitely sheds light on the preferences and experiences of the participants in their contact with the municipalities, an important insight for municipality professionals and inspectors. By involving them during the whole project, pain points become clear straight away and even point out issues that inspectors did not even think of or thought of in a different way. The experiences of the participants made the inspectors aware of the experiential knowledge that had never been revealed in a conventional inspection. As a result of the project the municipalities started to improve their services and evaluated their improvements with the client organization. And last but not least, the unexpected side effect was the huge impact on the participants themselves: they gained more self-esteem, they felt being taken seriously and valued and felt empowered by participating in this project.

However, the results of this project were merely small and practical improvements in accessibility rather than long term changes. Long term improvements in for instance the structural use of easy language or more personal contact require more attention than a mystery guest visit alone.

**Reflections/critical perspective: comment critically on the study reflecting on the things that went well and those that did not, so others can learn from this experience**

It is evident that involving experts by experience in supervision works when the user group is specific and distinctive, and also the topic of supervision is specified, for instance a specific theme. It is particularly suitable for supervision methods without existing minimum standards of safe and responsible care, or compliance with rules and laws, such as the ‘mirror without judgment’ methods of supervision. Also inspectors need to be open and motivated to try new methods such as the user perspective as central point for supervision, instead of their own regular methods of (enforcement) supervision.

Using the ‘mystery guests’ -method is not suitable in every supervision project. This method requires a large time investment, for the municipalities, the coaches and the inspectors, and trust, effort and patience in working together with a specific client group. In future mystery guest projects it is recommended to work with a real support request instead of a fictive one, for both the credibility of the participants and their ‘case’. This adds weight to the experiences of the participants and will be taken more seriously by the professionals.

The most important learning point from this project was that it is necessary to keep a close eye on the impact and (legal) consequences of a mystery guest visit for the professionals.

And last but not least: this project had a huge impact on the participants themselves. They gained more self-esteem, they felt being taken seriously and valued and felt empowered by participating in this project. It is therefore necessary that inspectorates in future projects value their participation by financial rewards and for instance a certificate.
