# Supplementary material for: The value of experts by experience in social domain supervision in the Netherlands: results from a ‘mystery guests’ project
Source: BMC Health Serv Res. 2024 Feb 9;24:187. doi: 10.1186/s12913-024-10692-y (PMC10858591; doi:10.1186/s12913-024-10692-y)
Supplement: Supplementary file 2 — Supplementary Material 2 [file 12913_2024_10692_MOESM2_ESM.pdf]

May 22 2018

Topic list for interviews with experts by experience in five municipalities:

The purpose of the interview is to ask about the experiences of the experts with the project. In accordance with the research proposal, two research questions are central:

1) How has the client perspective been shaped in this project?

2) How did the experts by experience the interpretation of the client perspective?

The following step-by-step plan was used to compile the questionnaire:

1) Consider which aspects of the research should be covered;

2) Get relevant questions from literature or use existing questions;

3) Turn all ideas into questions;

4) Organize the questions into clusters;

5) Check whether the research questions are covered;

6) Create a logical order;

7) Formulate an introduction and conclusion to the interview;

8) Test the instrument and adjust if necessary.

For the various research questions, we have come up with questions for each part of the way in which the clients are involved in the project. We also looked at the questions that have already been asked by the Inspectors in the evaluation interviews that were held in each municipality with the experts by experience and the questions that were already included in the research proposal.

In the evaluation interviews conducted by the Inspectors with the experts, the following questions were asked to the experts, according to the reports of these meetings:

- *What did you think of being a mystery guest?*
- *Is it also possible without a coach?*
- *How did you feel about being a guide on the road?*
- *What did the employees do well and what could be improved? **
- *What did you think of your experiences, what was good and what tips would you like to give the municipality?**

* These questions are not relevant to our evaluation research and will therefore not be used to formulate a topic list for interviews in the context of our research.

With regard to the way in which the client perspective has been shaped in this project, the following elements can be distinguished, these elements are reflected in the questions that have been formulated:

1) Experts by experience had a discussion with the inspectorate about whether it was a feasible idea to use experts by experience as mystery guests and what the conditions would be for this. JISD also spoke to the director of the LFB for this purpose.

2) Experts by experience have contributed to the formulation of the assessment framework with regard to accessibility. They were asked what is important to them in terms of accessibility to the social domain. This resulted in a list of assessment criteria against which accessibility to help and support from the municipality was assessed.

3) Experts by experience themselves (some with help) formulated help questions to test a number of different parts of the Social Domain for accessibility.

4) Experts by experience assessed the websites of the five municipalities in group meetings

5) 16 experts have been mystery guests, most have had group training, some have been individually prepared for their work as mystery guests.

6) Experts by experience have contacted the municipalities regarding their request for help and, in consultation with the DHYI, determined how they would do this (by e-mail/telephone or via a physical visit to the municipality, or all three);

7) The experts then gave back their experiences to the Inspectors and then discussed their findings directly in a meeting with the employees of the municipalities;

8) The Inspector provided feedback on these experiences at management level in a meeting with management; the experts were not present.

9) On January 11, 2018, there was a final meeting at which the experts were awarded a certificate.

10) Finally, a supervision report was drawn up with the experiences of the experts. This report has been written in such a way that it is also accessible to people with intellectual disabilities. The report was presented to the city councilor, sometimes with the press and experts present

In order to best meet the specific needs of the experts by experience, the interviews are focused on the specific involvement and role of the expert by experience in the project. Because the project took place a year ago, the interviewer will ask the expert by experience as much as possible during the interview about what his or her role was at the time.

The interview for the municipality of XXX is used as an example.

Interview format XXX; tailored to the specific experience of the interviewee

Introduction interview

Explanation of who I am and what I will do with this interview. I say that we are curious about the experiences with the project to see whether the Inspectorate can be helped more often by clients with an intellectual disability.<br><br>The interview lasts approximately half an hour. I explain that I will ask some questions for each part of the project. I will first provide a brief summary of the part of the project that the questions are about. Then I tell you what I'm going to ask questions about, and then I ask the questions. I look and listen carefully to see if you have understood my question correctly. If you do not understand a question properly, please let me know. It's been a long time, so if you don't remember, that's okay too. If you want to take a break in between, that is also possible. Let me know. At the very end of the interview I will also ask your coach a few questions.

Role of the client in the project:

1. How did you feel about participating in the inspection's project?
2. What did you do (meeting with requirements, assessed the website with a group, received training, played a mystery guest, provided feedback to the municipality, were present when presenting the final report to the alderman/press? Think about criteria for accessibility?)

At the start of the project, you were able to think about what you thought was important regarding the accessibility of the municipality. I'm going to ask some questions about this now:

3. How did you feel about being able to think about what you found important in your contact with the municipality in the beginning?
4. Did you find it difficult or easy to come up with points?
5. Was your input carefully listened to during this meeting?
6. When considering the points that you consider important in contact with the municipality, have you also used previous experiences you have had? Can you give an example?
7. How did you come up with this list? What was your input?
8. What do you think of the list that was created? Are there any things missing here?
9. Do you think that someone without an intellectual disability, such as an Inspector, could also come up with this list? If yes, why, if no, why not?
10. Did you enjoy thinking about this together in a group?
11. What did you like or dislike about this meeting?

You participated in assessing the municipality's websites during a number of group meetings. I'm going to ask you some questions about this:

12. How did you feel about participating in these meetings?
13. What did you like and dislike about these meetings?
14. Were you clear in advance what you had to do?
15. Were you well supervised?

16. Do you think this is a good way to assess the website? Why or why not?
17. How did you feel about assessing the websites in groups?
18. Do you think it is important that people with intellectual disabilities assess the website themselves? Why or why not?
19. Do you think that you, as a person with an intellectual disability, assess the website differently than, for example, an inspector? If so what do you think the difference would be?

You also went to the municipality with a self-made request for help. You had figured out that you were pregnant and didn't want this. You didn't dare tell your parents. You didn't really know what to do. You went to the municipality's walk-in consultation hours with this question. Your coach came with you. I am now going to ask a few questions about this visit to the walk-in consultation of the municipality:

20. How are you prepared to do something as a Mystery guest? How did you like that?
21. How did you feel about having to play a role?
22. How did you feel about your coach coming along? Why?
23. If you had gone alone, would things have been different?

After you visited the municipality, there were discussions with the Inspector. In these conversations you could tell what you thought of your contact with the municipality. Now I'm going to ask some questions about your conversations with the inspector:

24. <u>Evaluation of the results:</u>
25. How did you find the conversations with the Inspector?
26. Did the Inspector listen to you? Can you give an example of this?
27. Did you dare to say everything you wanted in these conversations? Why or not?
28. There has been no discussion with municipal employees about your experiences, how do you feel about that?
29. What would you have wanted to say if there had been a conversation?
30. Do you think the municipality will do something with your experiences? Why?

A report has now been written by the Inspectorate. That report has also been presented. I'm now going to ask some questions about this report.

31. <u>Evaluation of the report of the JISD</u>
32. Have you read the Inspectorate's report?
33. Do you understand what is in the report?
34. Do you think the report matches what you told the Inspector?
35. Were you also present when the report was presented? How did you like that?

The project is now finished. I'm very curious what you thought of this project. We now come to the end of the interview. Finally, I'd like to ask some questions about what you thought of the project.

36. <u>Evaluation of the project /final questions</u>
37. What did you think of the project? Which parts of the project did you like and dislike?
38. What was not arranged properly or properly in the various parts of the project?
39. Would you participate in this project again?

40. Would you recommend others with intellectual disabilities to participate in such a project? Why or why not?
41. If you were to participate again or recommend others to participate, what must be properly arranged in a project?

We have now come to the end of the interview, thank you very much for your cooperation and your time. Finally, I would like to ask your coach a few questions.

<u>Questions for the coach:</u>

- How did you experience the project?

- Do you have any tips or points for improvement regarding this project?

- Do you think other organizations could also work with experts by experience? If so, which organizations are you thinking of?

****
