# Supplementary material for: The value of experts by experience in social domain supervision in the Netherlands: results from a ‘mystery guests’ project
Source: BMC Health Serv Res. 2024 Feb 9;24:187. doi: 10.1186/s12913-024-10692-y (PMC10858591; doi:10.1186/s12913-024-10692-y)
Supplement: Supplementary file 3 — Supplementary Material 3 [file 12913_2024_10692_MOESM3_ESM.pdf]

May 22 2018

Topic list for interviews with municipality professionals

The purpose of the interview is to find out how public servants and service managers of the municipalities experienced the experts by experience in this project. The central research question according to the research proposal is:

How did the various participants (people with intellectual disabilities, inspectors, professionals working at the municipalities) experienced the interpretation of the client perspective in this project?

Two people from each municipality are interviewed. An employee who was present at the employee meeting and a service manager. The employee and the manager are interviewed separately so that they can talk freely.

Questions for the public servants:

Last year, a number of clients with a disability reported to the municipality with a self-made request for help to assess the extent to which the Social Domain is accessible to this target group. In addition, clients have contributed ideas about the implementation of the assessment framework with regard to accessibility and assessed websites.

1. What has been your involvement in this project?
2. Have you been approached by an expert yourself?
3. How did you experience the efforts of the experts in this project?
4. Are you aware that experts by experience are involved in this project in different ways? They have acted as a mystery guest, but also assessed websites and devised requirements for accessible access? What do you think of this?
5. What do you think about people with intellectual disabilities reporting to the municipality with a made-up request for help?
6. Do you think you should be informed in advance that such an investigation will be conducted in your municipality? If yes why, if no why not?
7. How do you feel that experts by experience, rather than the inspector, determined what the requirements for accessible access were?
8. Do you think that the input from the experts reveals issues that would otherwise not have come to light? If so can you give an example, if not why not?
9. The experiences of the mystery guests were fed back directly in a meeting. How did you experience this meeting?
10. What do you remember from this meeting?
11. Have there been any issues that surprised you that could not have been presented in any other way?
12. How do you view the effectiveness of such a meeting with experts by experience compared to a visit or feedback from an inspector?
13. Do you think that what was discussed in the meeting with the experts by experience is sufficiently reflected in the Inspectorate's report? if not what are you missing?
14. What experiences did you have with supervision beforehand?
15. What do you think of the use of experts by experience in general as an instrument for supervision?
16. What is the significance of this project for you as an employee? And for the municipality?
17. Do you think that the use of experts by experience could also be a suitable instrument on other themes within the social domain or in other sectors? Which?

Questions for the service managers:

1. What experience did you have with supervision beforehand?

2. What did you think of this project?

3. How did you experience the use of experts by experience in this project?

4. What has been the impact of this project with experts by experience on the organization? In terms of tips for the working methods and emotions of employees?

5. Do you think that you should be informed in advance that such a project will be conducted in your municipality? Why or why not?

6. What is the impact of the employee meeting on the employees in your estimation?

7. How did the meeting with feedback from the Inspectorate go?

8. What did you think of this meeting?

9. How do you see your input during that meeting reflected in the report?

10. What have the experiences of the experts taught you as a manager?

11. Can you comment on the points that emerged from this project, why or why not?

12. Do you think that the input from the experts reveals issues that would otherwise not have come to light? If so can you give an example, if not why not?

13. What has the municipality gained from the suggestions that employees themselves came up with in discussions with the experts? With what, what not and why?

14. What is the significance of this project for you as a manager and for the municipality in your view?

15. What do you think about the fact that the report was written in easy language and based on the experiences of people with intellectual disabilities?

16. Were you at the meeting to present the report to the councilor?

17. What did you think of one of the experts presenting the report to the councilor?

18. Does this project yield more for the municipality than if no experienced experts had participated? Why not?

19. Do you think the use of experienced experts as an instrument for supervision should also be followed in other sectors or other areas? Why or why not? Which areas?

20. What will you do in the future with the use of experienced experts?
