# Supplementary material for: The value of experts by experience in social domain supervision in the Netherlands: results from a ‘mystery guests’ project
Source: BMC Health Serv Res. 2024 Feb 9;24:187. doi: 10.1186/s12913-024-10692-y (PMC10858591; doi:10.1186/s12913-024-10692-y)
Supplement: Supplementary file 4 — Supplementary Material 4 [file 12913_2024_10692_MOESM4_ESM.docx]

**Interview guide inspectors**

• What are your activities within the JISD?

• What was your role in this project?

• How did you experience this project?

*Experts by experience:*

• What is the significance of the use of experts by experience for your actions as an inspector?

• What does this method (use of experts by experience) achieve?

• Does this approach lead to different types of information than if you do not involve experts by experience? Can you explain why or why not?

• How do you view the shelf life of experienced experts, does this make a difference for people with intellectual disabilities?

• What do you think is the value of using experts by experience for the JISD?

• How do you see the role of the expert by experience in the implementation of the JISD assessment framework? Do you think this has been done properly in this project? Why, why not?

• Has the content of the assessment framework changed due to the input of experienced experts? Why, why not? If so, give an example?

*Role of the inspectorate:*

• What is the role of the inspectorate in a project like this? Creating conditions or really actively arranging everything? And how does this relate to remote supervision in the social domain?

• What is the role of the inspectorate in the social domain? Has this influenced the way this project was carried out, if so how?

• Strengthening the position of clients with intellectual disabilities is also an effect of this project; does this fit within the tasks of the inspection in your view? Why, why not?

*Collaboration with stakeholders involved*:

• How did the cooperation with the municipalities proceed? Points of improvement?

• How do you explain the difference in drive/cooperation between the different municipalities?

• How did the collaboration with LFB go?

• What is the significance of involving an interest group, such as the LFB, in this project?

• As an inspector, do you need certain skills for the success of a project where you work with clients with intellectual disabilities? Or does this make no difference compared to using experts by experience without it?

*Mystery guest part of the project*:

• Do you think the use of mystery guests in supervision is permitted? Can you explain why?

• Must municipalities or other organizations that are subject to supervision be informed in advance of the use of mystery guests? Why, why not?

• Do you believe that the use of mystery guests provides different information than when you, as an Inspector, assess accessibility yourself? Why do you think?

• Is this a way to put the citizen's perspective at the center of supervision? Why or why not?

• Is this a more effective method of supervision, in terms of bringing about changes in the municipality? Why or why not?

• Is this an instrument that is also suitable in other care sectors? Why or why not, and what do you think about it?

Do you have any tips or points for improvement for a next project with experts by experience?

******
